# Target identification of usnic acid in bacterial and human cells†

**DOI:** 10.1039/d4cb00040d

**Published:** 2024-05-07

**Authors:** Stuart A. Ruddell, Dietrich Mostert, Stephan A. Sieber

**Affiliations:** a Center for Functional Protein Assemblies, Department of Bioscience, TUM School of Natural Sciences, Technical University of Munich Ernst-Otto-Fischer-Straße 8 85748 Garching Germany stephan.sieber@tum.de

## Abstract

Usnic acid is a natural product with versatile biological activities against various organisms. Here, we utilise a chemical proteomic strategy to gain insights into its target scope in bacterial and human cells. First, we excluded DNA binding as a major reason for its antibacterial activity, and second, we commenced with target profiling, which unravelled several metal cofactor-dependent enzymes in both species indicating a polypharmacological mode of action. Interestingly, our synthetic studies revealed a selectivity switch at usnic acid, which maintains antibacterial activity but lacks strong cytotoxic effects.

## Abstract 2

Activity-based probes of the natural product usnic acid reveal protein targets in bacterial and human cells.

## Graphical abstract

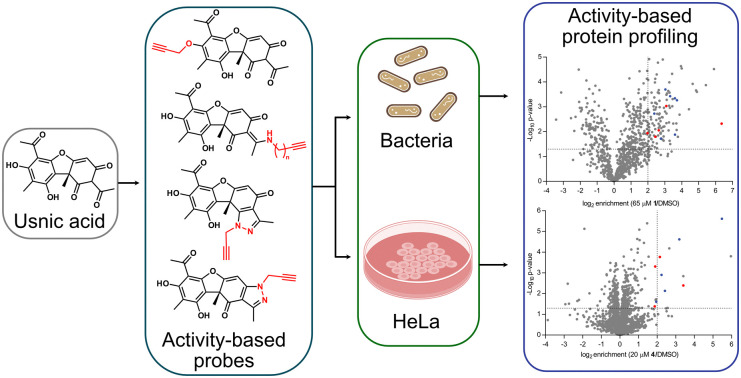

## Introduction

Antimicrobial resistance is a major global health concern caused in part by a lack of drugs with novel modes of action (MoAs). Most currently used antibiotics address a limited number of biological targets with long-established resistance mechanisms.^1,2^

Natural products (NPs) have evolved with living organisms to target distinct molecular pathways and, as such, have been used by humankind for thousands of years, from traditional folk medicine through the antibiotic golden age to modern-day drug discovery.^3,4^ Indeed, from 1981 to 2016, 60% of all FDA-approved antibiotic drugs were NPs, NP derived or NP mimicking – clearly displaying their continued importance in medicine.^5^

Many NPs, while never becoming approved clinical drugs, have been reported in the literature to possess antibacterial activities. However, comprehensive data as to their biological MoA is often lacking. This represents an opportunity to mine the literature in search of these compounds and to use state-of-the-art technologies to elucidate the cellular MoAs in search of novel druggable biological pathways without already established resistance mechanisms.

**Scheme 1:**
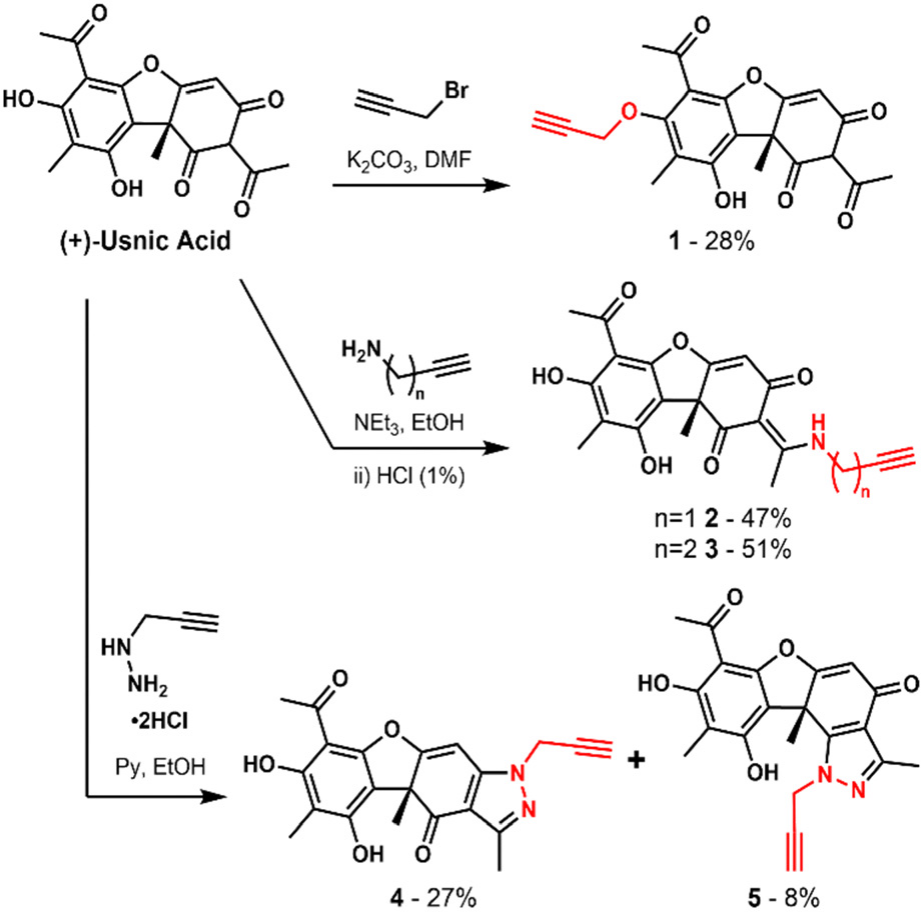
Structure of (+)-usnic acid and synthetic routes to alkyne derivatives (1–5) for ABPP target engagement studies.

Usnic acid, a lichen-derived natural product, represents one such compound (Scheme 1). It was first isolated in 1844, and since then, numerous scientific papers have been published reporting its diverse bioactivities.^6,7^ The most noteworthy for this study are its activities against Gram-positive bacteria, particularly *Enterococci*, *Staphylococci* and *Streptococci*, as well as cytotoxicity towards most human cell lines.^8–12^

Regarding the biological targets of usnic acid, several publications exist claiming different MoAs, including but not limited to decoupling of oxidative phosphorylation, inhibition of cytochrome enzymes, inhibition of RNA and DNA synthesis, interaction with DNA, DNA damage and a potent irreversible inhibition of 4-hydroxyphenylpyruvate dioxygenase (HPPD) in plants.^13–18^ The majority of reported protein interaction targets of usnic acid are metal cofactor dependent. As usnic acid is known to bind metals and exhibits several chelating moieties, general metal binding could be a possible explanation for the seemingly high promiscuity.^19–21^ Additionally, target promiscuity could be explained by putative covalent binding to cysteine or other nucleophilic amino acid residues *via* its electrophilic Michael acceptor moiety.

In any case, these differing reported activities suggest a more complex reality than a single target protein and thus indicate a probable polypharmacological mechanism of action; however, to date, no proteomic approach has been applied to usnic acid to elucidate its interaction partners at the proteome level in living cells in an unbiased manner.

## Results and discussion

In order to assess covalent protein targets, we chemically introduced alkyne handles at various positions into the usnic acid core structure as biorthogonal tags for use in activity based addition were rationally chosen to evaluate the requirement of specific functional groups of usnic acid, and to maximise the likelihood of achieving an active probe.^22^ Probe 1 was derivatised at the phenolic position by alkylation with propargyl proteomic profiling (ABPP) (Scheme 1).^23–25^ The sites of alkyne bromide, affording a probe with the triketone moiety intact. Based on an analogous reported synthetic strategy, probes 2 and 3 were derivatised as an enamine, by condensation of usnic acid with either propargylamine or 3-butynylamine respectively.^10^ The final two probes, 4 and 5, were derivatised as a pyrazole ring following a related synthetic strategy.^12^ Here, propynylhydrazide hydrochloride was condensed with usnic acid, generating a separable mixture of the two regioisomeric probes. With the five probes in hand, evaluation of their bioactivities was conducted in both bacterial and human cells.

To assess bacterial susceptibility to each of the synthesised probes, MIC (minimum inhibitory concentration) data was evaluated in 10 strains of pathogenic bacteria (Table 1). Like usnic acid itself, none of the probes were active against the tested Gram-negative strains. In accordance with previous literature, usnic acid was most active against Gram-positive *Streptococcal* (3–6 μM), *Enterococcal* (6–13 μM) and *Staphylococcal* (25 μM) bacteria, including multidrug resistant strains.^8,10,15^ The two most active probes, 1 and 4 (anti-*Streptococcal* activity: 31–62 μM) closely followed this trend, albeit with a slight reduction of activity (within 1 order of magnitude) as is often observed for probe derivatives.^22^ While Probe 5 had some slight activity (anti-*Streptococcal* activity: 125–250 μM), it was classified with probe 2 and 3 as inactive (anti-*Streptococcal* activity: >250 μM), due to its significant drop in activity as compared to usnic acid itself.

**Table 1:** Bioactivities of usnic acid and its synthesised alkyne derivatives against multiple bacterial strains and human HeLa cell line

	Bacterial strain/human cell line	UA	1	2	3	4	5
MIC (μM)	*Streptococcus pneumoniae* DSM20566	3	31	>250	>250	62	125
	*Streptococcus mutans* UA159	6	62	>250	>250	62	250
	*Enterococcus faecalis* V583 (VRE)	6	62	>250	>250	62	250
	*Enterococcus faecium* DSM20477 (VSE)	10	62	>250	>250	125	250
	*Enterococcus faecium* DSM17050 (VRE)	13	62	>250	>250	125	250
	*Staphylococcus aureus* ATCC29213 (MSSA)	25	125	>250	>250	>250	>250
	*Staphylococcus aureus* USA300 (MRSA)	25	125	>250	>250	250	250
	*Escherichia coli* 536	>100	—	—	—	—	—
	*Pseudomonas aeruginosa* PAO1	>100	—	—	—	—	—
	*Acinetobacter baumannii* DSM30007	>100	—	—	—	—	—

CC_50_ (μM)	HeLa	27	206	11	19	12	20

To assess human toxicity, MTT cell viability assays were conducted (Table 1).^26^ Here, in accordance with literature, usnic acid had a half maximal cytotoxic concentration (CC_50_) of 27 μM against the HeLa cell line.^12,27^ Interestingly, probes 2, 3, 4 and 5 (3 of which were inactive against bacterial cells) where all slightly more cytotoxic than usnic acid, and the most toxic probe to bacterial cells (1) was significantly less toxic to human cells. As this is the only probe to be functionalised at one of the phenolic positions, this suggests it to be a significantly important moiety for human toxicity, and that derivatisation at this position could act as a switch to maintain bacterial activity, while reducing human side effects.

**Fig. 1:**
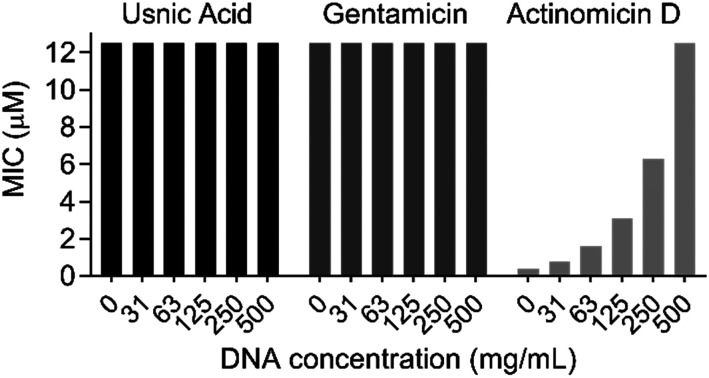
DNA binding MIC shift assay with gentamicin (negative control), actinomycin D (positive control) and usnic acid against *E. faecium* DSM17050.

**Fig. 2:**
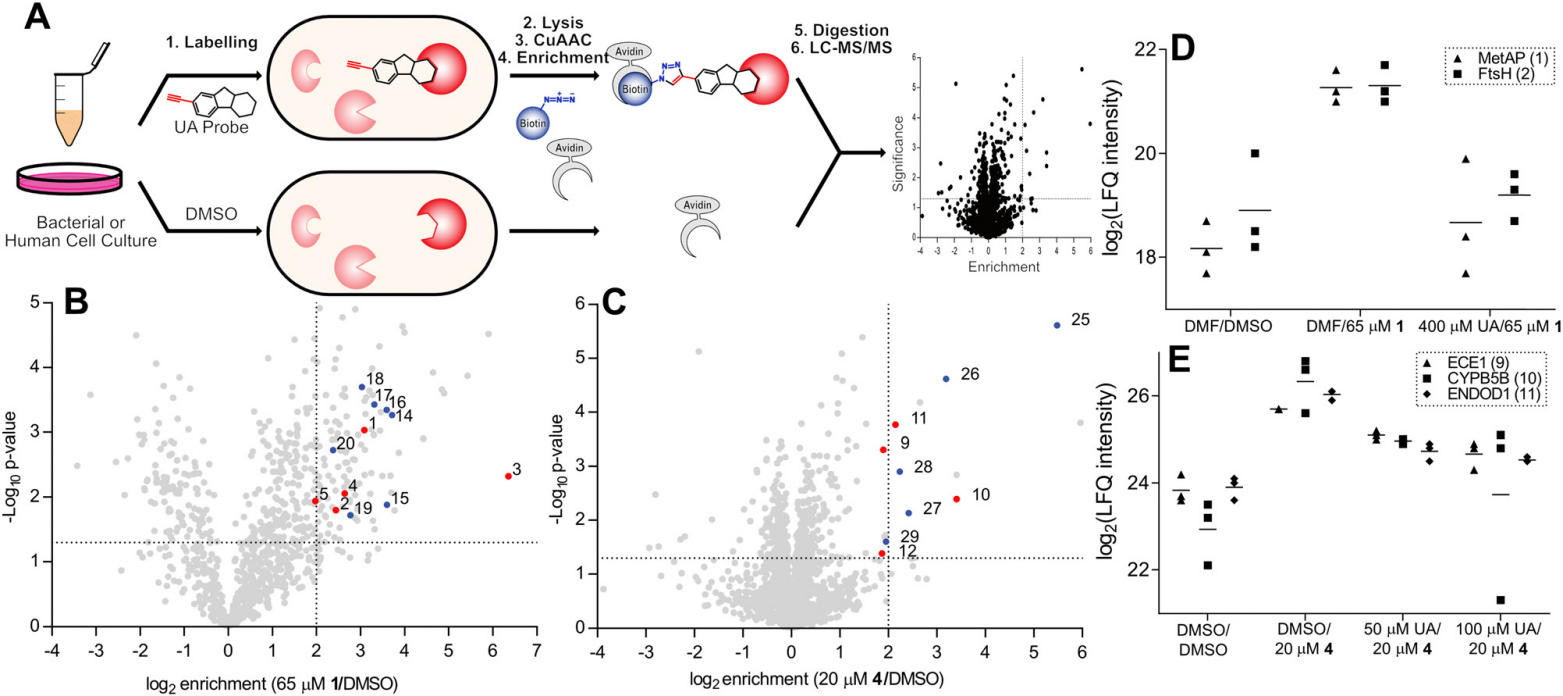
(A) ABPP workflow involving labelling of whole cells with alkyne probe, followed by cell lysis and CuAAC chemistry to rhodamine or biotin azide. Rhodamine-labelled proteins are separated using SDS-PAGE and fluorescently visualised. Biotin-labelled proteins are enriched with avidin beads, the proteins enzymatically digested, and the resulting peptides analysed by liquid chromatography coupled tandem mass spectrometry (LC-MS/MS). (B) and (C) Volcano plots depicting probe enrichment against significance for Probe 1 in *E. faecium* DSM17050 (B) and probe 4 in HeLa cells (C). Significance thresholds; *p*-value < 0.05, enrichment – ×4 above DMSO background. Numbered and coloured – significantly enriched and outcompeted, red – metal cofactor protein, blue – unknown or non-metal cofactor. Additional enrichment and competition volcano plots are available (Fig. S2, ESI†) and numbered proteins are listed (Table 2 and Table S1, ESI†). (D) and (E) Corresponding profile plots depicting select enriched and outcompeted metal binding proteins, identified in volcano plots B and C for probe 1 in *E. faecium* DSM17050 (D) and probe 4 in cells (E). UA = usnic acid; MetAP = methione aminopeptidase; DMF = dimethylformamide.

Before conducting extensive proteomic investigation, the reports of usnic acid as a DNA-interacting agent were further investigated, as this would be consistent with its planar polycyclic aromatic structure.^15,16,28–30^ Biophysical measurements were previously used to elucidate an interaction between usnic acid and DNA, and while this concluded usnic acid to likely bind DNA *via* surface interactions, we further investigated its effect on antibacterial activity. To this end, we optimised a DNA binding MIC shift assay, to assess if the interaction with DNA is the primary cause of toxicity.^31–33^ Using gentamicin as a negative control and the known DNA intercalator actinomycin D as a positive control, we could clearly conclude that DNA binding does not play a significant role in the antibacterial mechanism of action of usnic acid (Fig. 1 and Fig. S1, ESI†). Target identification was performed using an ABPP approach to elucidate the protein interaction partners of usnic acid.^26,34–38^ In short, live cells (bacterial or human) were labelled with either the alkyne probe or DMSO (negative control) followed by cell lysis (Fig. 2A). Copper catalysed alkyne azide click chemistry (CuAAC) was performed to click labelled proteins to either a rhodamine azide dye (for analysis using SDS-PAGE) or a biotinazide affinity tag for avidin bead enrichment.^39,40^ The enriched proteins were enzymatically digested (trypsin) and the resultant peptides analysed by LC-MS/MS using label free quantification (LFQ) data analysis.^41^ To exclude non-specific binding and enrichment, competition experiments were performed using the same workflow, with an additional pre-incubation of competitor (either usnic acid or DMSO as negative control).

ABPP labelling was initially assessed *via* gel-based analysis using probe 1 in the multidrug resistant *Enterococcal* strains *E. faecium* DSM17050 and *E. faecalis* V583, which depicted strong concentration dependant labelling confirming a covalent attachment to protein targets (Fig. S2A and B, ESI†). Using the most active probes in their corresponding organisms – namely, probe 1 in *E. faecalis* V583 and *E. faecium* DSM17050 and probes 4 and 2 in human HeLa cells – optimal probe concentrations were selected and quantitative enrichment studies were conducted (Fig. 2B and C and Fig. S2C–H, ESI†). Furthermore, competitive LC/MS-MS labelling with various excesses of usnic acid was performed to confirm the significantly enriched proteins as specific binders of usnic acid (Fig. 2D, E, Table 2 and Fig. S2C–H, Table S1, ESI†).

**Table 2:** Enriched and outcompeted metal cofactor-dependant proteins from all active probes. [M] – unspecified or multiple metal ions accepted, MP – metalloprotease, OR – oxidoreductase. Complete list of enriched and outcompeted proteins available in ESI (Table S1). Proteins on grey background can be found in supplementary volcano plots (Fig. S2, ESI)

Probe	Organism		Protein/gene name	Metal	Role
1	*E. faecium* DSM17050	1	Methionine aminopeptidase	[M]	MP
		2	FtsH	Zn	MP
		3	Cytochrome P450	Fe	OR
		4	Cd translocating ATPase	[M]	
		5	Cation transporter	[M]	
	*E. faecalis* V583	6	Gelatinase	Zn	MP
		7	Oxidoreductase	Fe	OR
		8	Coproporphyrin III ferrochelatase	Fe	

4	HeLa	9	Endothelin converting enzyme 1	Zn	MP
		10	Cytochrome B5B	Fe	OR
		11	ENDOD1	[M]	
		12	Na-coupled neutral AA transporter	Na	

2	HeLa	9	Endothelin converting enzyme 1	Zn	MP
		13	Heme oxygenase 2	Fe	

Targets of particular note include two essential metalloproteases – methionine aminopeptidase (1) and FtsH (2) – which were strongly enriched as well as competed by the parent molecule.^42,43^ Additionally, gelatinase (6) – a zinc metalloprotease known to be important for *E. faecalis* virulence and biofilm formation was strongly enriched and outcompeted. Endothelin converting enzyme 1 (ECE1) (9), a zinc metalloprotease known to be important for cancer cell invasiveness was enriched with strong competition in human labelling by both probes.^44,45^ Moreover, several cytochrome oxidoreductases (3, 7, 10) were also identified, both in bacteria and human labelling. In total, 13 proteins known to contain metal ion cofactors were enriched across all proteomes and probes used (Table 2).

To investigate this further, one of the top hits from the labelling in *E. faecium*, the methionine aminopeptidase (1) was cloned and purified and labelling was analysed *in vitro*. The purified protein was incubated with the activity based probes and clicked to rhodamine-azide, to allow assessment of labelling after SDS-PAGE *via* fluorescence (Fig. S3, ESI†). The protein was specifically labelled by probe 1, the probe which enriched the MetAP in the ABPP experiments, validating those results. The antibacterially inactive probe 5 showed significantly weaker labelling compared to the active probe 1. Interestingly, the probe used for labelling in human cells (probe 4), revealed similarly weak labelling as probe 5. There was no difference in labelling between the protein with the addition of CoCl_2_, or without which indicates that direct metal complexation is not the sole reason for protein binding in this case.

While some of the identified targets are not essential, the consistent enrichment and competition of metal binding proteins, in particular; oxidoreductases and metalloproteases, is nonetheless compelling as many of the literature-described targets and mechanisms of action for usnic acid contain proteins with metal cofactors.^14,18,21^ Thus, this study highlights usnic acid as a promiscuous metalloprotein binding natural product and provides the first comprehensive inventory of its human and bacterial target proteins.

## Conclusions

In summary, five alkyne probes rationally derivatised at various positions of usnic acids core scaffold were synthesised and used for ABPP target identification to elucidate its protein interaction profile. Strong enrichment and competition was observed for multiple proteins in various organisms indicating a broad reactivity, with noteworthy preference of metalloproteases and oxidoreductases. Additionally, while usnic acid is reported to interact with DNA, we determined this interaction to be inconsequential to its bacterial toxicity. Therefore, we conclude that usnic acid exhibits its toxic mechanism through a polypharmacological MoA, with particular affinity for metal cofactor dependant proteins. Interestingly, alkylation of one of the phenolic position of usnic acid turned out to be an important selectivity switch which reduced toxicity against human cells while maintaining its antibacterial properties. This intriguing finding could be pursued further by derivatisation of the other phenol group or other constituents of the aromatic ring and measuring the differential effect on human toxicity and antibacterial activity. This molecule thus represents a promising starting point for further antibiotic development.

## Data availability

The mass spectrometry proteomics data have been deposited to the ProteomeXchange Consortium *via* the PRIDE^46^ partner repository with the dataset identifier PXD049013.

## Conflicts of interest

There are no conflicts to declare.

## Supplementary Material

CB-005-D4CB00040D-s001
