# Supplementary material for: Target identification of usnic acid in bacterial and human cells
Source: RSC Chem Biol. 2024 May 7;5(7):617–21. doi: 10.1039/d4cb00040d (PMC11221533; doi:10.1039/d4cb00040d)
Supplement: CB-005-D4CB00040D-s001 [file CB-005-D4CB00040D-s001.pdf]

Electronic Supplementary Material (ESI) for RSC Chemical Biology.
This journal is © The Royal Society of Chemistry 2024

Supplementary Information

DNA binding assay

Gentimicin (μM)

	100	50	25	12.5	6.25	3.125	1.5625	0.78125	0.390625	0
DNA 500 μg/mL	0.0507	0.0492	0.0505	0.0511	0.2242	0.31810001	0.3339	0.3547	0.37310001	0.38769999
	0.0495	0.0489	0.0493	0.0504	0.27270001	0.2737	0.40450001	0.35030001	0.44299999	0.45989999
DNA 250 μg/mL	0.0487	0.0486	0.0492	0.0498	0.2406	0.35620001	0.35839999	0.36790001	0.4269	0.4386
	0.0495	0.0482	0.0485	0.0477	0.2509	0.3838	0.38139999	0.35030001	0.41620001	0.48640001
DNA 125 μg/mL	0.0493	0.0513	0.0499	0.0524	0.2431	0.39309999	0.3527	0.36399999	0.4339	0.38850001
	0.0478	0.0487	0.0482	0.0495	0.0973	0.40009999	0.41890001	0.39390001	0.4497	0.3741
DNA 63 μg/mL	0.0484	0.0502	0.0484	0.0516	0.14300001	0.34810001	0.3154	0.373	0.3759	0.38389999
	0.0479	0.0486	0.0488	0.0484	0.1098	0.57990003	0.52999997	0.32159999	0.32170001	0.389
DNA 31 μg/mL	0.0554	0.0482	0.0487	0.0493	0.1121	0.59689999	0.54259998	0.5837	0.44769999	0.3655
	0.0481	0.0485	0.0481	0.0482	0.31349999	0.52640003	0.542	0.5959	0.449	0.3461
DNA 0 μg/mL	0.0484	0.0481	0.0485	0.0491	0.13259999	0.47960001	0.4914	0.58359998	0.64770001	0.3915
	0.0492	0.0481	0.0492	0.0496	0.1565	0.4682	0.55379999	0.63690001	0.59859997	0.6548

Actinomycin D (μM)

	100	50	25	12.5	6.25	3.125	1.5625	0.78125	0.390625	0
DNA 500 μg/mL	0.0461	0.0461	0.0455	0.0467	0.4756	0.48429999	0.38069999	0.59280002	0.51950002	0.63630003
	0.0451	0.0447	0.0491	0.048	0.25150001	0.4673	0.45379999	0.58840001	0.42269999	0.40689999
DNA 250 μg/mL	0.0454	0.0462	0.0462	0.0454	0.0465	0.38190001	0.47780001	0.60549998	0.32960001	0.60720003
	0.0452	0.0448	0.0515	0.0439	0.0458	0.2314	0.37529999	0.62550002	0.50809997	0.53960002
DNA 125 μg/mL	0.0458	0.0441	0.0452	0.0448	0.0458	0.046	0.44670001	0.64920002	0.38890001	0.54549998
	0.046	0.0445	0.0449	0.0449	0.0453	0.0458	0.39449999	0.5801	0.48210001	0.45410001
DNA 63 μg/mL	0.0467	0.0455	0.0453	0.0455	0.0461	0.0459	0.0492	0.34419999	0.31189999	0.39340001
	0.0461	0.0452	0.0457	0.0448	0.0457	0.045	0.068	0.252	0.3536	0.2994
DNA 31 μg/mL	0.0456	0.0452	0.0456	0.049	0.0484	0.0493	0.0449	0.0467	0.41580001	0.40009999
	0.0452	0.0449	0.0447	0.0443	0.052	0.0453	0.0458	0.0462	0.2457	0.35600001
DNA 0 μg/mL	0.0449	0.0544	0.0488	0.0456	0.0449	0.0447	0.0446	0.0451	0.0462	0.41150001
	0.0455	0.0451	0.0449	0.046	0.0449	0.0446	0.0439	0.0458	0.0458	0.2929

Usnic acid (μM)

	100	50	25	12.5	6.25	3.125	1.5625	0.78125	0.390625	0
DNA 500 μg/mL	0.0455	0.0452	0.0447	0.0456	0.1208	0.1665	0.18080001	0.2445	0.2561	0.56029999
	0.0548	0.0445	0.0453	0.044	0.1268	0.1716	0.1939	0.3811	0.4357	0.4944
DNA 250 μg/mL	0.0472	0.0453	0.0447	0.045	0.1203	0.15700001	0.34819999	0.2642	0.37959999	0.55650002
	0.0448	0.0435	0.044	0.0435	0.1071	0.1485	0.1727	0.23100001	0.3854	0.551
DNA 125 μg/mL	0.0491	0.0439	0.0437	0.045	0.1391	0.1487	0.2009	0.29480001	0.4109	0.48980001
	0.0502	0.0451	0.0445	0.0459	0.1201	0.1469	0.1839	0.27320001	0.52899998	0.33160001
DNA 63 μg/mL	0.0499	0.0453	0.0452	0.0683	0.1322	0.15440001	0.2079	0.2167	0.28169999	0.3154
	0.0457	0.0453	0.0456	0.0446	0.1229	0.1496	0.1865	0.2024	0.34529999	0.3364
DNA 31 μg/mL	0.0453	0.0459	0.0457	0.0456	0.13860001	0.14480001	0.25009999	0.1793	0.32589999	0.4377
	0.0447	0.0452	0.0453	0.0444	0.1243	0.1486	0.19220001	0.18000001	0.29789999	0.33739999
DNA 0 μg/mL	0.0459	0.0458	0.045	0.0455	0.13249999	0.1435	0.19850001	0.197	0.3669	0.3432
	0.0475	0.0446	0.0446	0.0456	0.126	0.1167	0.17569999	0.1927	0.2577	0.31549999

	MIC (μM)		
DNA (μg/mL)	Actinomicin D	Gentamicin	Usnic acid
0	0.4	12.5	12.5
31	0.8	12.5	12.5
63	1.6	12.5	12.5
125	3.1	12.5	12.5
250	6.3	12.5	12.5
500	12.5	12.5	12.5

Figure S1. DNA binding assay results as OD_{600} values. Gentimicin negative control (no DNA intercalation known) showed no deviation of MIC against *E. faecium* DSM17050 upon external DNA treatment. Actinomicin D positive control for intercalation[1] showed dose dependant increase of MIC upon increasing external DNA concentration. Usnic acid showed no alteration of MIC upon external DNA treatment – similar to gentamicin (negative control for DNA intercalation).

Supplementary ABPP data.

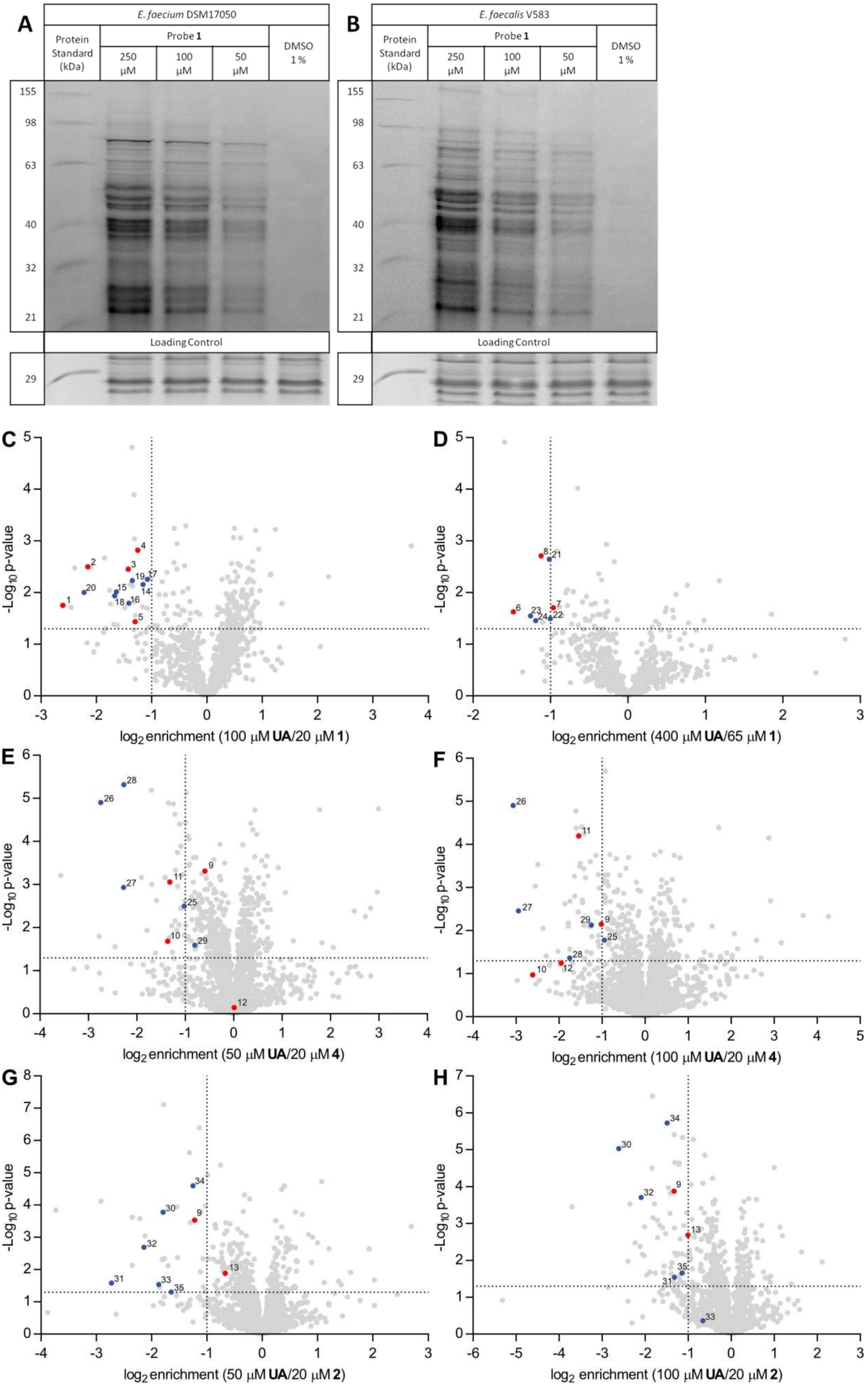

Figure S2. A+B) Analytical ABPP gels of probe **1** against both *E. faecium* DSM17050 (A) and *E. faecalis* V583 (B) showing dose dependant labelling. C–H) Preparative ABPP usnic acid competition volcano plots against probe **1** in *E. faecium* DSM17050 (C)

and *E. faecalis* V583 (D), probe **4** in Hela cells at x2.5 (E) and x5 (F) competitor and probe **2** in Hela cells at x2.5 (G) and x5 (H) competitor. Lines indicates Significance thresholds; p-value < 0.05, competition (enrichment) – x2 below DMSO background. Numbered and coloured – significantly enriched and outcompeted, Red – Metal cofactor protein, Blue – Unknown or non-metal cofactor. Proteins listed in Table S1.

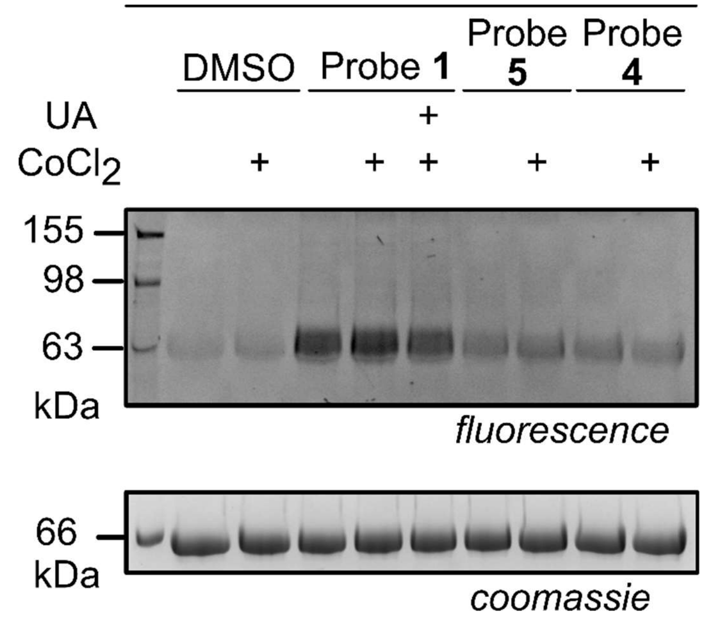

Figure S3. Labelling of purified *E. faecium* Methionine Aminopeptidase (MetAP). The MetAP was cloned and expressed and purified as a fusion protein with N-terminal maltose binding protein (MBP) for easier expression and purification. The respective probes were incubated with the protein and later clicked to rhodamine azide to allow detection of labelling via fluorescence. The protein concentration for the assay was 5 µM. The probe concentration was 25 µM, and the usnic acid (UA) concentration was 200 µM. Probe **1** strongly labels the protein, validating the result from the ABPP analysis. There is no clear difference in labelling between protein with and without $CoCl_2$. The experiment was conducted in two independent replicates.

Table S1. List of all enriched and outcompeted proteins identified by probes **1** (*E. faecium* DSM17050 and *E. faecalis* V583), **4** (HeLa) and **2** (HeLa).

Probe	Organism		Protein/Gene Name/Uniprot Code	Metal	Role
**1**	E. faecium DSM17050	1	Methionine aminopeptidase	[M]	MP
		2	ATP-dependent zinc metalloprotease FtsH	Zn	MP
		3	Cytochrome P450	Fe	OR
		4	Cd translocating ATPase	[M]	
		5	Cation diffusion facilitator family transporter	[M]	
		14	Phosphomevalonate kinase	-	-
		15	2-dehydropantoate 2-reductase	-	-
		16	ABC transporter ATP-binding protein	-	-
		17	GNAT family N-acetyltransferase	-	-
		18	Glutathione peroxidase	-	-
		19	YtxH domain-containing protein	-	-
		20	BcrR	-	-
	E. faecalis V583	6	Gelatinase	Zn	MP
		7	Oxidoreductase	Fe	OR
		8	Coproporphyrin III ferrochelatase	Fe	
		21	Q834V8 – Uncharacterized Protein	-	-
		22	Transcriptional regulator, GntR family	-	-
		23	Glycosyl transferase, group 1 family protein	-	-
		24	Glycerol-3-phosphate dehydrogenase [NAD(P)+]	-	-
**4**	HeLa	9	Endothelin converting enzyme 1	Zn	MP
		10	Cytochrome B5B	Fe	OR
		11	Endonuclease domain-containing 1 protein	[M]	
		12	Na-coupled neutral AA transporter	Na	
		25	Reticulon-3	-	-
		26	2-hydroxyacylsphingosine 1-beta-galactosyltransferase	-	-
		27	Gamma-tubulin complex component 6	-	-
		28	Mitochondrial pyruvate carrier 2	-	-
		29	hydroxysteroid dehydrogenase-like protein 1	-	-
**2**	HeLa	9	Endothelin converting enzyme 1	Zn	MP
		13	Heme oxygenase 2	Fe	
		30	Reticulon-3	-	-
		31	A0A5C2G655 – Uncharacterized Protein	-	-
		32	Membralin	-	-
		33	WD repeat-containing protein 82	-	-
		34	Carnitine O-palmitoyltransferase 1	-	-
		35	Polymerase delta-interacting protein 2	-	-

General Methods

All reagents were purchased from commercial suppliers and used without further purification unless otherwise stated. Reactions involving air-sensitive agents and dry solvents were performed in glassware that had been dried in an oven (150 °C) or flame-dried *in vacuo* and allowed to cool *in vacuo* before being flushed with argon. These reactions were carried out with the exclusion of air using an argon atmosphere. Reactions were monitored by thin layer chromatography (TLC) on Merck silica gel 60 covered aluminium sheets. TLC plates were developed under UV-light and/or with an acidic ethanolic anisaldehyde solution or a $KMnO_4$ solution. NMR spectra were recorded on a Bruker AVHD-400 spectrometer (^1H NMR at 400 MHz, ^{13}C NMR at 100 MHz) or a Bruker AVHD-500 spectrometer (^1H NMR at 500 MHz and ^{13}C NMR at 125 MHz). Chemical shifts are reported in ppm. ^1H NMR spectra were recorded with $CDCl_3$ as the solvent using residual $CHCl_3$ (δ = 7.26) as internal standard, and for ^{13}C NMR spectra the chemical shifts are reported relative to the central resonance of $CDCl_3$ (δ = 77.16). Signals in NMR spectra are described as singlet (s), doublet (d), triplet (t), quartet (q), multiplet (m), broad (br) or a combination of these, which refers to the spin-spin coupling pattern. Spin-spin coupling constants are reported in Hertz (Hz) and are uncorrected. Two-dimensional NMR spectroscopy (COSY, HSQC, HMBC) were used where appropriate to assist the assignment of signals in the ^1H and ^{13}C NMR spectra. High resolution mass spectra were recorded under ESI conditions using a Thermo Scientific LTQ Orbitrap XL.

Chemical synthesis of usnic acid probes

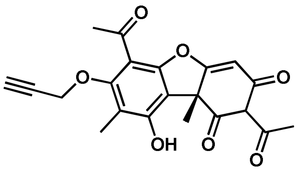

(9b*R*)-2,6-diacetyl-9-hydroxy-8,9b-dimethyl-7-(prop-2-yn-1-yloxy)dibenzo[*b*,*d*]furan-1,3(2*H*,9b*H*)-dione (**1**).

To a stirred suspension of usnic acid (300 mg, 0.90 mmol, 1 equiv.) and potassium carbonate (300 mg, 2.1 mmol, 2.5 equiv.) in dimethylformamide (2.0 mL, 0.5 M) was added propargyl bromide (95 µL, 1.0 mmol, 1.1 equiv.) and the resultant suspension was stirred at room temperature for 16 hours. To this was added dichloromethane (20 mL) and water (20 mL) and the phases separated. The aqueous phase was extracted with dichloromethane (3 x 20 mL) and the combined organic phases were then washed with half saturated aqueous sodium chloride solution (3 x 20 mL), dried over magnesium sulphate and evaporated *in vacuo* resulting in a yellow solid. The crude material was then purified by column chromatography on silica gel using an eluent of dichloromethane to 5% ethyl acetate in dichloromethane to afford the title compound **1** (96 mg, 0.25 mmol, 28%) as a yellow solid.

^1H NMR (400 MHz, $CDCl_3$) δ 10.88 (1H, s), 5.94 (1H, s), 4.57 (2H, d, *J* = 2.4), 2.64 (3H, s), 2.61 (3H, s), 2.53 (1H, d, *J* = 2.4), 2.20 (3H, s), 1.77 (3H, s). ^{13}C NMR (100 MHz, $CDCl_3$) δ 201.9, 198.0, 196.7, 191.8, 180.0, 157.0, 155.1, 153.0, 117.3, 113.1, 109.2, 105.3, 98.2, 78.3, 76.7, 62.3, 59.3, 32.4, 32.1, 28.1, 9.4. HRMS (ESI) exact mass calculated for $C_{21}H_{19}O_7$ $[M+H]^+$ m/z 383.1125, found m/z 383.1124.

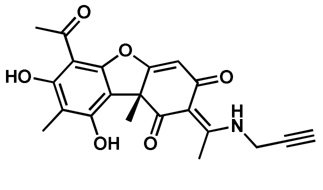

(*R*,*E*)-6-acetyl-7,9-dihydroxy-8,9b-dimethyl-2-(1-(prop-2-yn-1-ylamino)ethylidene)dibenzo[*b*,*d*]furan-1,3(2*H*,9b*H*)-dione (**2**).

Prepared according to a modified literature procedure [2]. To a stirred solution of usnic acid (0.20 g, 0.60 mmol, 1 equiv.) in ethanol (3.0 mL, 0.2 M) was added triethylamine (0.10 mL, 0.76 mmol, 1.3 equiv.) and propargylamine (42 µL, 0.64 mmol, 1.1 equiv.) and the resulting solution was stirred at 80 °C for 2.5 hours then cooled to room temperature. Aqueous hydrochloric acid (5 mL, 0.3 M) was added and the reaction stirred at room temperature for 18 hours. The resulting precipitate was collected, washed with cold aqueous hydrochloric acid (10 mL, 0.3 M) and water (10 mL) then dried *in vacuo*. Purification by column chromatography on silica gel using an eluent of 5% ethyl acetate in dichloromethane afforded title compound **2** (0.11 g, 0.28 mmol, 47%) as a yellow solid.

^1H NMR (400 MHz, $CDCl_3$) δ 13.46 (1H, s), 13.31 (1H, s), 11.69 (1H, s), 5.76 (1H, s), 4.22 (2H, dd, *J* = 5.5, 2.6), 2.67 (3H, s), 2.62 (3H, s), 2.44 (1H, t, *J* = 2.5), 2.04 (3H, s), 1.66 (3H, s). ^{13}C NMR (100 MHz, $CDCl_3$) δ 200.7, 198.7, 190.2, 175.7, 174.2, 163.5,

158.2, 155.9, 108.1, 105.0, 102.8, 102.5, 101.4, 76.5, 74.3, 57.3, 33.3, 32.0, 31.3, 18.4, 7.5. HRMS (ESI) exact mass calculated for $C_{21}H_{20}NO_6$ [M+H]$^+$ m/z 382.1285, found m/z 382.1285.

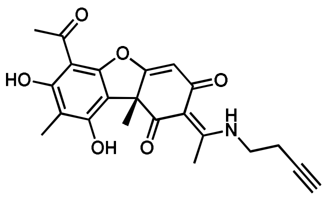

(*R*,*E*)-6-acetyl-2-(1-(but-3-yn-1-ylamino)ethylidene)-7,9-dihydroxy-8,9b-dimethyldibenzo[*b*,*d*]furan-1,3(2*H*,9b*H*)-dione (**3**).

Prepared according to a modified literature procedure [2]. To a stirred solution of usnic acid (0.20 g, 0.60 mmol, 1 equiv.) in ethanol (3.0 mL, 0.2 M) was added triethylamine (0.10 mL, 0.76 mmol, 1.3 equiv.) and propargylamine (42 µL, 0.64 mmol, 1.1 equiv.) and the resulting solution was stirred at 80 °C for 3 hours then cooled to room temperature. Aqueous hydrochloric acid (5 mL, 0.3 M) was added and the reaction stirred at room temperature for 18 hours. The resulting precipitate was collected, washed with cold aqueous hydrochloric acid (10 mL, 0.3 M) and water (10 mL) then dried *in vacuo*. Purification by column chromatography on silica gel using an eluent of 5% ethyl acetate in dichloromethane afforded title compound **3** (0.12 g, 0.31 mmol, 52%) as a yellow solid.

^{1}H NMR (400 MHz, $CDCl_3$) δ 13.51 (1H, s), 13.34 (1H, s), 11.82 (1H, s), 5.81 (1H, s), 3.67 (2H, dt, *J* = 6.3, 6.3), 2.65 (6H, s), 2.62 (2H, td, *J* = 6.6, 2.5), 2.16 (1H, t, *J* = 2.5), 2.07 (3H, s), 1.69 (3H, s). ^{13}C NMR (100 MHz, $CDCl_3$) δ 200.8, 198.6, 190.3, 175.3, 174.3, 163.6, 158.3, 156.0, 108.1, 105.1, 102.5, 102.5, 101.5, 79.3, 71.9, 57.3, 42.7, 32.1, 31.4, 19.4, 18.5, 7.6. HRMS (ESI) exact mass calculated for $C_{22}H_{22}NO_6$ [M+H]$^+$ m/z 396.1442, found m/z 396.1442.

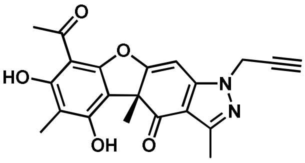

4

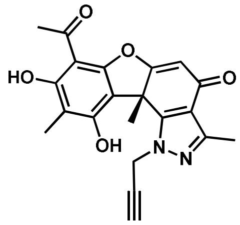

5

(*R*)-8-acetyl-5,7-dihydroxy-3,4a,6-trimethyl-1-(prop-2-yn-1-yl)-1,4a-dihydro-4*H*-benzofuro[3,2-*f*]indazol-4-one (**4**)
(*S*)-7-acetyl-8,10-dihydroxy-3,9,10b-trimethyl-1-(prop-2-yn-1-yl)-1,10b-dihydro-4*H*-benzofuro[2,3-*g*]indazol-4-one (**5**).

Prepared according to a modified literature procedure [3] A stirred solution of prop-2-yn-1-ylhydrazine hydrochloride (130 mg, 0.96 mmol, 1.1 equiv.) and pyridine (76 mg, 0.96 mmol, 1.1 equiv.) in ethanol (5 mL, 0.2 M) was heated at 70 °C for 10 mins, followed by the addition of usnic acid (300 mg, 0.90 mmol, 1.0 equiv.). The resultant suspension was heated at reflux for 3 hours, before addition of water (5 mL) and dichloromethane (20 mL.). The phases were separated, the aqueous extracted with dichloromethane (3 x 15 mL) and the combined organic phases dried over magnesium sulphate and evaporated *in vacuo* resulting in a yellow solid. The crude material was then purified by column chromatography on silica gel using an eluent of dichloromethane to 2% ethyl acetate in dichloromethane to afford the title compounds **4** (93 mg, 0.25 mmol, 28%) and **5** (26 mg, 0.069 mmol, 8%) as yellow solids.

4. ^{1}H NMR (400 MHz, $CDCl_3$) δ 13.34 (1H, s), 11.07 (1H, s), 6.29 (1H, s), 4.93–4.89 (2H, m), 2.67 (3H, s), 2.54–2.51 (1H, m), 2.49 (3H, s), 2.09 (3H, s), 1.73 (3H, s). ^{13}C NMR (100 MHz, $CDCl_3$) δ 200.5, 195.9, 173.1, 163.7, 157.8, 156.4, 150.8, 149.0, 110.6, 108.5, 104.1, 101.7, 87.9, 75.9, 75.3, 60.5, 39.5, 31.4, 30.7, 13.3, 7.6. HRMS (ESI) exact mass calculated for $C_{21}H_{19}N_2O_5$ [M+H]$^+$ m/z 379.1289, found m/z 379.1287.

5. ^{1}H NMR (400 MHz, $CDCl_3$) δ 13.49 (1H, s), 6.01 (1H, s), 5.68 (1H, dd, *J* = 17.5, 2.4), 5.33 (1H, dd, *J* = 17.5, 2.4), 2.74 (3H, s), 2.54 (3H, s), 2.35 (1H, t, *J* = 2.3), 2.16 (3H, s), 1.87 (3H, s). ^{13}C NMR (100 MHz, $CDCl_3$) δ 201.5, 182.3, 178.6, 163.0, 158.1, 155.6, 149.9, 147.4, 116.2, 106.6, 105.9, 105.6, 102.6, 78.5, 73.8, 47.7, 41.7, 31.8, 31.4, 13.6, 7.7. HRMS (ESI) exact mass calculated for $C_{21}H_{19}N_2O_5$ [M+H]$^+$ m/z 379.1289, found m/z 379.1287.

Biochemical Methods

Bacterial Culture

Unless otherwise stated all Gram-negative strains (*E. coli*, *A. baumannii* and *P. aeruginosa*) were cultivated using sterile (autoclaved) LB medium (Lysogeny Broth; 10 g/L casein peptone, 5 g/L sodium chloride, 5 g/L yeast extract, pH 7.5 in dd H_2O) with 200 rpm shaking at 37 °C. For the culture of all streptococcal strains (*S. mutans* and *S. pneumoniae*), THB (Todd–Hewitt Broth; 10 g/L casein peptone, 10 g/L meat peptone, 3.1 g/L heart infusion, 2.5 g/L sodium carbonate, 2 g/L dextrose, 2 g/L sodium chloride 0.4 g/L disodium phosphate, pH 7.8 in dd H_2O) was used at 37 °C and (for *S. pneumoniae*) shaking at 200 rpm. For *S. mutans* no shaking was used and additionally 5% CO_2 was required. For the cultivation of all enterococcal and staphylococcal strains (E. faecalis, E, faecium and S. aureus) BHI medium (Brain Heart Infusion; 7.5 g/L brain infusion, 10 g/L heart infusion, 10 g/L casein peptone, 5 g/L sodium chloride, 2.5 g/L disodium phosphate, 2 g/L glucose, pH 7.4 in dd H_2O) was used with 200 rpm shaking at 37 °C. For overnight cultures, 5 mL of fresh medium was inoculated (1:1000) with bacterial cryostock using a sterile pipette tip into a sterile plastic culture tube. The culture was then incubated at 37 °C with 200 rpm shaking for 16 hours.

Human Cell Culture

HeLa cell line was cultivated in sterile high glucose DMEM (Dulbecco's Modified Eagle Medium, 4.5 g/L glucose) supplemented with 10% (v/v) heat inactivated FBS (Foetal Bovine Serum) and 2 mM L-glutamine at 37 °C in a humidified atmosphere containing 5% CO_2. Cells were split upon reaching 90% confluency, wherein the media was removed by suction, the adherent cells washed with sterile (autoclaved) PBS solution, detached with accutase® and diluted with fresh growth medium to a cell density of 1 x 10^6 cells/mL

Minimum Inhibitory Concentration (MIC)

Transparent flat bottomed 96 well plates were prepared by filling the outermost wells with 200 µL of fresh media to remove edge/evaporation effects and function as a sterile control. To conduct serial dilution of test compounds, 2 µL of a x100 DMSO solution of test compound was added to the first well. Then 100 µL of medium was added to the first well (with the compound) and 50 µL of medium to every other well. The first well was then mixed by pipetting up and down followed by transfer of 50 µL of the solution to the next well. This was repeated until the end of the row, discarding 50 µL from the final transferred well and leaving two wells with no compound added. To one of these was added 1 µL DMSO (as a vehicle control) and the other well was left with only medium as a growth control. To each well was added 50 µL of bacterial suspension generated from the inoculation (1:10,000) of fresh medium from fresh overnight culture. All compounds were assessed in triplicate and plates were incubated at 37 °C with 200 rpm shaking for 16 hours. The plates were then measured by eye and using a Tecan M Nano+ microplate reader to measure the optical density at 600 nm (OD_{600}). The MIC was determined to be the lowest concentration at which no bacterial growth (turbidity of solution) was detectable.

MTT Cell Viability Assay

Transparent flat bottomed 96 well plates were prepared by filling the outermost wells with 200 µL of fresh media to remove edge/evaporation effects and function as a sterile control. The remaining wells were seeded with 4000 cells per well and the cells allowed to reattach for 16 hours in 200 µL of fresh medium (37 °C, 5% CO_2). A compound serial dilution plate was then prepared for ease of compound transfer to the test plate. Here the outermost wells were also not utilised. 2 µL of a x100 DMSO solution of test compound was added to the first well. Then 100 µL of medium (without FCS) was added to the first well (with the compound) and 50 µL of medium (without FCS) to every other well. The first well was then mixed by pipetting up and down followed by transfer of 50 µL of the solution to the next well. This was repeated until the end of the row, discarding 50 µL from the final transferred well and leaving two wells with no compound added. To one of these was added 1 µL DMSO (as a vehicle control) and the other well was left with only medium as a growth control. To complete preparation of the compound plate, 50 µL of fresh media (without FCS) was added to each well, to a final volume of 101 µL. All compounds were tested in triplicate. The media was then removed from the seeded test plate, and the corresponding wells of the compound plate transferred via multichannel pipette to the test plate. The cells were then incubated for 24 hours (37 °C, 5% CO_2). To quantify viable cells, 20 µL of a 5 mg/mL stock solution (PBS) of 3-(4,5-dimethylthiazol-2-yl)-2,5-diphenyltetrazolium bromide (MTT) was added to each well and cells then incubated for an additional 2 hours (37 °C, 5% CO_2). Medium was then removed and the metabolised substrate was dissolved in 200 µL DMSO and quantified using a Tecan M Nano+ microplate reader (absorbance: λ = 570 nm, reference wavelength: λ = 630 nm). Metabolic activity of compound treated cells was determined by comparison with the DMSO treated cells and half maximal cytotoxic concentrations (CC_{50}) were calculated using the log(inhibitor) *vs.* response – variable slope (four parameters) function in GraphPad Prism 5.

DNA Binding Assay

To assess the effect of DNA binding on the bactericidal effect of usnic acid, previously described DNA binding assays were optimised. (10.1039/C9RA06844A) (10.1128/AAC.03132-14). In short, the test compound was pre incubated with varying

concentrations of external DNA (Salmon sperm) and the MIC evaluated. DNA intercalation manifests as an increase to the compounds MIC with increasing DNA concentrations, and with a lack of DNA intercalation, the compounds MIC remains unaffected by increasing DNA concentrations. Transparent flat bottomed 96 well plates were prepared by filling the outermost wells with fresh media (200 µL) to remove edge/evaporation effects and function as a sterile control. Compound titration (100 µM–390nM) was conducted across the plate (2x test concentration, 50 µL), while external salmon DNA was titrated (500 µg/mL–31 µg/mL) orthogonally down the plate (4x test concentration, 25 µL), with no compound (1%DMSO) vehicle control and no DNA negative controls included and all concentration combinations conducted in duplicate. The plate was then incubated for 1 hour (37 °, 200 rpm) before the addition of *E. faecium* DSM17050 suspension (25 µL) generated by inoculation (1:10,000) of fresh media from fresh overnight culture. Plates were incubated for 16 hours (37 °C, 200 rpm) and then measured using a Tecan M Nano+ microplate reader to measure the optical density at 600 nm (OD_{600}). The MIC was determined to be the lowest concentration at which no bacterial growth (turbidity of solution) was detectable. As positive control, the known DNA intercalator actinomycin D was used[1], and as negative control gentamicin was used. The results (OD_{600} values) are shown (Fig. S1) with bacterial growth highlighted in red. As expected, gentamicin shows no deviation to the MIC under the large concentration span of external DNA. The positive control actinomycin, known to intercalate DNA, has an MIC of 390 nM in the absence of external DNA. However dose dependant increase of the MIC was observed upon increase of DNA concentration up to a maximum tested MIC of 12.5 µM at 500 µg/mL DNA. Usnic acid showed no alteration of the MIC, similar to the negative control gentamicin.

Analytical Gel-Based ABPP Workflow

Fresh media was inoculated (1:100) with fresh overnight culture and cultured until stationary phase plus one hour (37 °C, 200 rpm) before being harvested (6000 g, 4 °C, 10 min), washed (2 x PBS, 6000 g, 4 °C, 10 min) and resuspended in PBS to a final OD_{600} of 40. 100 µL of the suspension was then aliquoted into Eppindorf tubes and then either 1 µL of a x100 DMSO stock of probe (final concentration of 1% DMSO) or 1 µL of DMSO (as negative control) was added and incubated for 1 hour (37 °C, 200 rpm). For competition experiments, an additional 1 hour preincubation (37 °C, 200 rpm) with either 1 µL of a x100 DMSO stock of competitor or 1 µL of DMSO (as negative control) was conducted. Suspensions were then harvested (6000 g, 4 °C, 10 min), washed (2 x PBS, 6000 g, 4 °C, 10 min) and resuspended in 100 µL PBS + 0.4 % sodium dodecyl sulphate (SDS). Lysis was conducted by sonication (4 x 30 sec, 80% intensity, Sonopuls HD 2070 ultrasonic rod, Bandelin electronic GmbH) with cooling breaks on ice. After cell lysis, the lysates was clarified by centrifugation (20,000 g, 30 min, 10 °C) and the supernatant transferred to fresh Eppindorf tubes. The protein concentration was measured using the Pierce BCA Protein Assay Kit (Thermo Fischer Scientific, Pierce Biotechnology) and samples were adjusted to the same concentration using PBS + 0.4% SDS. 88 µL of the standardised lysates were then subjected to the CuAAC reaction by addition of 12 µL of a click master mix [2 µL Rhodamine-azide (5 mM, DMSO stock), 2 µL TCEP (tris(2-carboxyethyl)phosphine) (52 mM, dd H_2O stock), 6 µL TBTA (Tris(benzyltriazolylmethyl)amine) (1.7 mM, 4:1 ^{t}BuOH:DMSO stock), 2 µL $CuSO_4$ (50 mM, dd H_2O stock)] and incubated at room temperature for one hour in the absence of light. To each sample was added 100 µL of x2 Lämmli buffer (Tris HCl 19.9 g/L, Glycerin 200 mL/L, SDS, 40 g/L, 2-mercaptoethanol 100 mL/L, Bromophenol blue 50 mg/L, dd H_2O) and samples were then heat treated at 95 °C for 15 mins. Heat treated samples were then separated by SDS-PAGE (12.5% polyacrylamide gel) and visualised with an ImageQuant LAS-4000 image reader (GE Healthcare) equipped with a Fujinon VRF43LMD3 lens and a 575DF20 filter (FujiFilm). The BenchMark Fluorescent Protein Standard (Thermo Fisher Scientific) and the Roti-Mark Standard (Carl Roth) were used as protein size standards. Loading control was assessed by coomassie staining (454 mL/L ethanol, 92 mL/L acetic acid, 2.5 g/L Coomassie Brilliant Blue R-250, dd H_2O) and subsequent destaining (200 mL/L ethanol, 100 mL/L acetic acid, dd H_2O).

Preparative MS Based ABPP Workflow

For the preparation of labelled bacterial lysates, fresh media was inoculated (1:100) with fresh overnight culture and cultured until stationary phase plus one hour (37 °C, 200 rpm) before being harvested (6000 g, 4 °C, 10 min), washed (2 x PBS, 6000 g, 4 °C, 10 min) and resuspended in PBS to a final OD_{600} of 40. 1000 µL of the suspension was then aliquoted into Eppindorf tubes and then either 10 µL of a x100 DMSO stock of probe (final concentration of 1% DMSO) or 10 µL of DMSO (as negative control) was added and incubated for 1 hour (37 °C, 200 rpm). For competition experiments, an additional 1 hour preincubation (37 °C, 200 rpm) with either 10 µL of a x100 DMSO stock of competitor or 10 µL of DMSO (as negative control) was conducted. All conditions were conducted either in triplicate or quadruplicate. Suspensions were then harvested (6000 g, 4 °C, 10 min), washed (2 x PBS, 6000 g, 4 °C, 10 min) and resuspended in 1000 µL PBS + 0.4 % sodium dodecyl sulphate (SDS). Lysis was conducted by sonication (4 x 30 sec, 80% intensity, Sonopuls HD 2070 ultrasonic rod, Bandelin electronic GmbH) with cooling breaks on ice. After cell lysis, the lysates was clarified by centrifugation (20,000 g, 30 min, 10 °C) and the supernatant transferred to fresh Eppindorf tubes. The protein concentration was measured using the Pierce BCA Protein Assay Kit (Thermo Fischer Scientific, Pierce Biotechnology) and samples were adjusted to the same concentration using PBS + 0.4% SDS.

For the preparation of labelled human cell lysates, cells were cultured in 15 mL dishes to 90% confluency. Media was then removed by suction, and cells washed twice with 10 mL PBS. To the cells was then added 10 mL of PBS containing either 100

µL of a x100 DMSO stock of probe (final concentration of 1% DMSO) or 100 µL of DMSO (as negative control) was added and incubated for one hour (37 °C, 5% CO_2). For competition experiments, an additional 1 hour preincubation (37 °C, 5% CO_2) with either 100 µL of a x100 DMSO stock of competitor or 100 µL of DMSO (as negative control) was conducted. All conditions were conducted either in triplicate or quadruplicate. Cells were then scraped from the dishes, pelletized (600 g, 5 min, 4 °C) and washed twice with 10 mL PBS (600 g, 5 min, 4 °C). Cells were then resuspended in lysis buffer (PBS + 1% NP40 and 1% sodium deoxycholate) and incubated on ice for 15 min followed by mild sonication (10% intensity, 15 sec, Sonopuls HD 2070 ultrasonic rod, Bandelin electronic GmbH) on ice. After cell lysis, the lysates was clarified by centrifugation (20,000 g, 30 min, 10 °C) and the supernatant transferred to fresh Eppindorf tubes. The protein concentration was measured using the Pierce BCA Protein Assay Kit (Thermo Fischer Scientific, Pierce Biotechnology) and samples were adjusted to the same concentration using PBS + 0.4% SDS.

From this point, labelled bacterial and human lysates were treated equally. 880 µL of the standardised lysates were subjected to the CuAAC reaction by addition of 120 µL of a click master mix [20 µL Biotin-PEG$_3$-azide (10 mM, DMSO stock), 20 µL TCEP (52 mM, dd H_2O stock), 60 µL TBTA (1.7 mM, 4:1 ^{t}BuOH:DMSO stock), 20 µL $CuSO_4$ (50 mM, dd H_2O stock)] and incubated at room temperature for one hour. Proteins were then precipitated by addition of 4 mL cold (–80 °C) acetone and incubation at –20 °C for 16 hours. Precipitated proteins were then pelletized (4000 g, 10 min), the supernatant removed and the pellet washed twice with 1 mL cold (–80 °C) methanol (20,000 g, 10 min, 4 °C) before dissolution in 500 mL PBS +0.4% SDS. For enrichment, the protein solutions were added to 50 µL of pre washed avidin-agarose bead suspension (1.1 mg/mL, washed thrice with PBS +0.4% SDS) and incubated at room temperature for one hour with constant mild agitation. To remove unbound proteins, beads were washed thrice with PBS +0.4% SDS, thrice with 6 M urea (dd H_2O) and thrice with PBS before resuspension in 200 µL capping buffer (7 M urea, 2 M thiourea in 20 mM HEPES). Proteins were reduced by addition of 2 µL TCEP (500 mM in dd H_2O) and incubation for one hour (37 C, 600 rpm) followed by alkylation by addition of 4 µL iodoacetamide (500 mM in 50 mM aqueous TEAB (Triethylammoniumbicarbonat)) and incubation in the absence of light (rt, 30 min, 600 rpm). Excess iodoacetamide was quenched by the addition of 4 µL DTT (Dithiothreitol)(500 mM in dd H_2O) and incubation (rt, 30 min, 600 rpm). For on-bead enzymatic digestion of the proteins, 600 µL TEAB (50 mM in dd H_2O) was added followed by 1.5 µL trypsin solution (0.5 µg/µL in 50 mM acetic acid) and incubation for 16 hours (37 °C, 600 rpm). To quench the digestion, 10 µL formic acid was added and the bead pelletized (20,000 g, 3 min, rt). The supernatant was subsequently loaded onto pre equilibrated (3 mL 0.1% aqueous trifluoroacetic acid) 50 mg SepPak C18 columns and the peptides washed thrice with 1 mL 0.1% aqueous trifluoroacetic acid and once with 500 µL 0.5% aqueous formic acid. Peptides were then eluted with 750 µL elution buffer (0.5% formic acid in 4:1 acetonitrile:dd H_2O) and samples concentrated to dryness and stored at –20 °C until LC-MS/MS measurement.

LC-MS/MS Measurement

Before measurement, peptides were dissolved in 30-45 µL 1% aqueous formic acid, sonicated for 15 minutes and filtered with 0.22 µm Ultrafree-MC centrifugal filters (Merck) (pre equilibrated with 1% aqueous formic acid). Samples were analysed using an UltiMate 3000 nano HPLC system (Dionex) equipped with an Acclaim C18 PepMap100 75 µm ID x 2 cm trap and Acclaim PepMap RSLC C18 (75 µm ID x 50 cm) separation columns in an EASY-spray setting coupled to a Q Exactive Plus (Thermo Fisher). Between 1-5 µL of filtered peptide solutions were injected on the trap column with a flow rate of 5 µL/min (0.1% aqueous trifluoroacetic acid) and followed by transfer to the separation column with a flow rate of 0.3 µL/min. Separation was performed with a 152 min gradient (buffer A: 0.1% aqueous formic acid, buffer B: 0.1% formic acid in acetonitrile) beginning with 0% buffer B in buffer A, increasing to 5% buffer B over seven min, then to 22% over the next 105 min, to 32% over the next ten min and to 90% over the next ten min. Buffer B was held at 90% for an additional 10 min before decreasing back to 5% over 0.1 min and held at 5% until the end of the run. Ionization was conducted at a spray voltage of 1.6 kV and capillary temperature of 275 °C. The Q Exactive plus was operated in a TopN data dependant mode of 12. Full scan acquisition was performed in the orbitrap mass analyser to a resolution of R=140,000 and an AGC target of 3.0 e^6 with a maximum injection time of 80 ms and a scan range set to 300-1,500 m/z. Dynamic exclusion duration was set to 60 ms. Isolation was performed in the quadrupole using a window of 1.6 m/z. Precursors with charge states >1, intensities higher than 1.0 e^5 and a minimum AGC target of 1.0 e^5 were selected for fragmentation. Peptide fragments were generated using higher energy collisional dissociation (HCD) with a normalized collision energy of 27%. MS2 scans were detected in the orbitrap at a resolution of 17,500 with and a maximum injection time of 100 ms.

Data Analysis

LC-MS/MS data was analysed using MaxQuant software (version 1.6.2.10)[4] with the following settings enabled: fixed modification- carbamidomethylation (Cys); variable modification- oxidation (Met), acetylation (N-term), NH (Asp); proteolytic enzyme- trypsin/P; missed cleavages- 2; main search tolerance- 4.5 ppm; MS/MS tolerance- 0.5 Da; false discovery rates- 0.01. Additionally, label free quantification (LFQ) was utilised[5], and the options "LFQ" and "match between runs" (0.7 min match and 20 min alignment time windows) were enabled. Searches were performed against the Uniprot databases for the respective organisms. Statistical analysis of the produced data was performed using the Perseus software (version 1.6.1.3).[6] Putative contaminants, peptides only identified by site and reverse peptides were filtered out. Data was filtered for either

two or three valid values in at least one group and missing values were imputed over the entire matrix. LFQ intensities were $-\log_2$ transformed and for statistical evaluation, $-\log_{10}$ p values were obtained by a two-sided two sample Student's *t*-test. The mass spectrometry proteomics data have been deposited to the ProteomeXchange Consortium via the PRIDE[7] partner repository with the dataset identifier PXD049013. The reviewers can access the data with the username reviewer_pxd049013@ebi.ac.uk and password 9EbXisrs.

Cloning and Expression of E. faecium MBP-MetAP

The map gene was amplified from the extracted genomic DNA of E. faecium DSM17050 using PCR and the primers in the following table:

Primer 1	ggggacaagtttgtacaaaaaagcaggctttgagaatctttattttcagggcATGATTACCTTAAAATCTAAAAGGGAAATTG
Primer 2	ggggaccactttgtacaagaaagctgggtgTTAATAAGTTCCTTCTTCTCCTTGAGATG

The resulting PCR fragment was purified using a preparative agarose gel and subsequent DNA gel extraction. The cleaned PCR fragment was then cloned into pETG41K (via pDONR207) using the Gateway BP and LR Clonase (Invitrogen) according to the standard manufacturer protocol. The plasmid was transformed into BL21 DE3 cells and grown on LB-kanamycin plates. Two colonies were picked, grown in LB-Kan and the plasmid extracted using a miniprep kit (Machery Nagel). The plasmid was sequenced and a colony with the correct sequence was used for protein Expression purification. The maltose binding protein (MBP)-TEV-Ef-MetAP fusion protein was expressed solubly at 18°C overnight. 1 L of LB-Kan were inoculated (1:100) with BL21 DE3 pETG41K_SaMetAP and grown to OD600 = 0.5. Protein expression was induced by adding IPTG to a final concentration of 0.5 mM. The next day, cells were harvested and washed with cold PBS. Cell pellets were reconstituted in lysis buffer (50 mM Hepes pH 7.5, 150 mM NaCl, 1 mM TCEP, 5% glycerol, 1 mM PMSF, small amount of DNASE) and lysed by sonication (7 min at 30%, 3 min at 60%, 7 min at 30% intensity, Sonopuls HD 2070 ultrasonic rod, Bandelin electronic GmbH). The lysate was cleared at 38.000 xg for 45 min and the supernatant was filtered through a 0.45 µm filter prior to purification using an Äkta Pure Protein Purification System (Cytiva). Due to the low binding capacity of the MBPTrap the purification was split into 5 runs (from 1 L harvested cells). Each time, 7 mL Lysate was loaded onto an equilibrated 5 mL MBPTrap column (Cytiva) at a flow-rate of 2 mL/min. The column was washed with 6 column-volumes (CV) buffer A (50 mM Hepes pH 7.4, 150 mM NaCl, 1 mM TCEP, 5% glycerol, 5 mM EDTA) at a flow-rate of 5 mL/min and eluted over 3 CVs with 100% buffer B (50 mM Hepes pH 7.4, 150 mM NaCl, 1 mM TCEP, 5 mM EDTA, 5% glycerol, 10 mM Maltose). The elution fractions were pooled (not concentrated) and desalted using 2 combined 5 mL HiTrap Desalting column (Cytiva) into buffer C (50 mM Hepes pH 7.4, 150 mM NaCl, 1 mM TCEP, 5% glycerol) to remove EDTA. The protein was further purified using a 5 mL HisTrapHP column (Cytiva) using the N-terminal His6 tag of MBP. For this, the sample was loaded and the column was washed with 9 column-volumes (CV) buffer C (50 mM Hepes pH 7.4, 150 mM NaCl, 1 mM TCEP, 5% glycerol) at a flow-rate of 5 mL/min and eluted over 5 CVs with 100% buffer D (50 mM Hepes pH 7.4, 150 mM NaCl, 1 mM TCEP, 5 mM EDTA, 5% glycerol, 500 mM Imidazole) at a flow-rate of 5 mL/min. The elution fractions were pooled (not concentrated) and desalted using 2 combined 5 mL HiTrap Desalting column (Cytiva) into buffer C (50 mM Hepes pH 7.4, 150 mM NaCl, 1 mM TCEP, 5% glycerol) to remove imidazole. MBP-Ef-MetAP was confirmed by IP-MS and SDS-PAGE and the fractions were pooled and concentrated.

Labelling of purified MBP-*Ef*-MetAP

An aliquot of the concentrated protein (348 µM) was thawed on ice. To half of the protein, $CoCl_2$ was added to an equimolar concentration from a 10 mM stock in LC-MS grade H_2O. The protein was incubated for 20 minutes at RT to allow binding of the metal to the protein. The protein with and without metal, were diluted to 5 µM in PBS with 25 µM of the respective probe and 200 µM usnic acid for the competition sample (50 µL total volume per sample). The samples were incubated for 30 minutes at 37 °C. To each sample 4.9 µL of a click master mix [0.6 µL Rhodamine-azide (20 mM, DMSO stock), 0.6 µL TCEP (tris(2-carboxyethyl)phosphine) (100 mM, dd H_2O stock), 2.5 µL TBTA (Tris(benzyltriazolylmethyl)amine) (1.7 mM, 4:1 ^{t}BuOH:DMSO stock), 1.2 µL $CuSO_4$ (50 mM, dd H_2O stock)] were added and incubated at room temperature for one hour in the absence of light. To each sample was added 55 µL of x2 Lämmli buffer (Tris HCl 19.9 g/L, Glycerin 200 mL/L, SDS, 40 g/L, 2-mercaptoethanol 100 mL/L, Bromophenol blue 50 mg/L, dd H_2O) and samples were then heat treated at 95 °C for 10 mins. SDS-PAGE and fluorescence readout were performed as described above.

NMR spectra

1

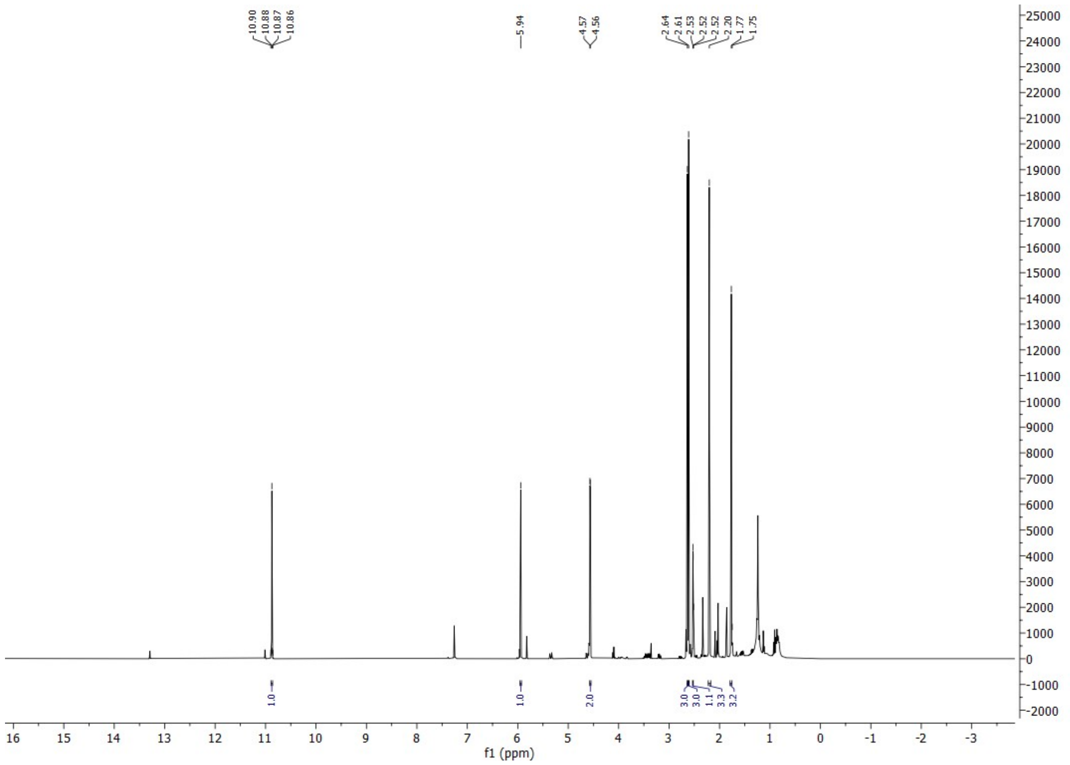

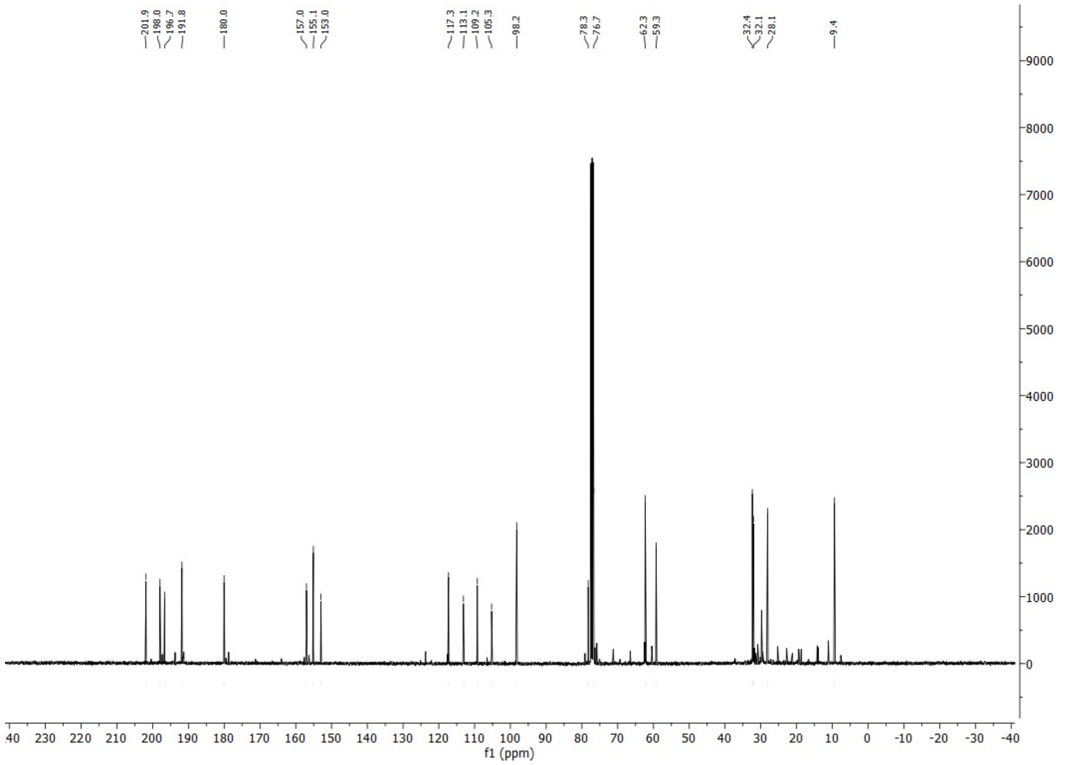

NMR-spectra of (9b*R*)-2,6-diacetyl-9-hydroxy-8,9b-dimethyl-7-(prop-2-yn-1-yloxy)dibenzo[*b*,*d*]furan-1,3(2*H*,9b*H*)-dione (**1**). Top spectra is ^{1}H NMR (400 MHz, $CDCl_3$) and bottom is ^{13}C NMR (100 MHz, $CDCl_3$).

2

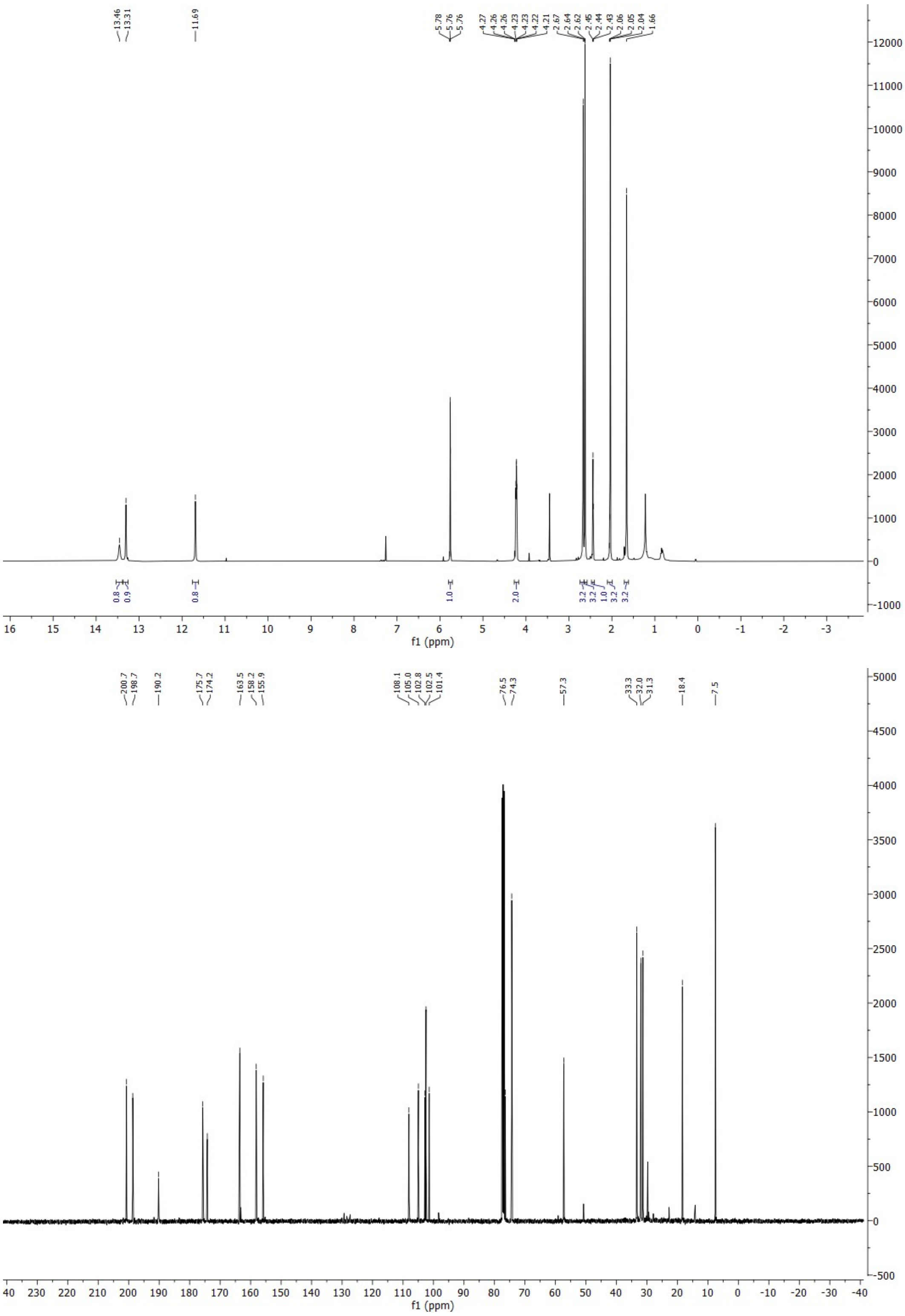

NMR-spectra of (*R*,*E*)-6-acetyl-7,9-dihydroxy-8,9b-dimethyl-2-(1-(prop-2-yn-1-ylamino)ethylidene)dibenzo[*b*,*d*]furan-1,3(2*H*,9b*H*)-dione (**2**). Top spectra is ^{1}H NMR (400 MHz, $CDCl_3$) and bottom is ^{13}C NMR (100 MHz, $CDCl_3$).

3

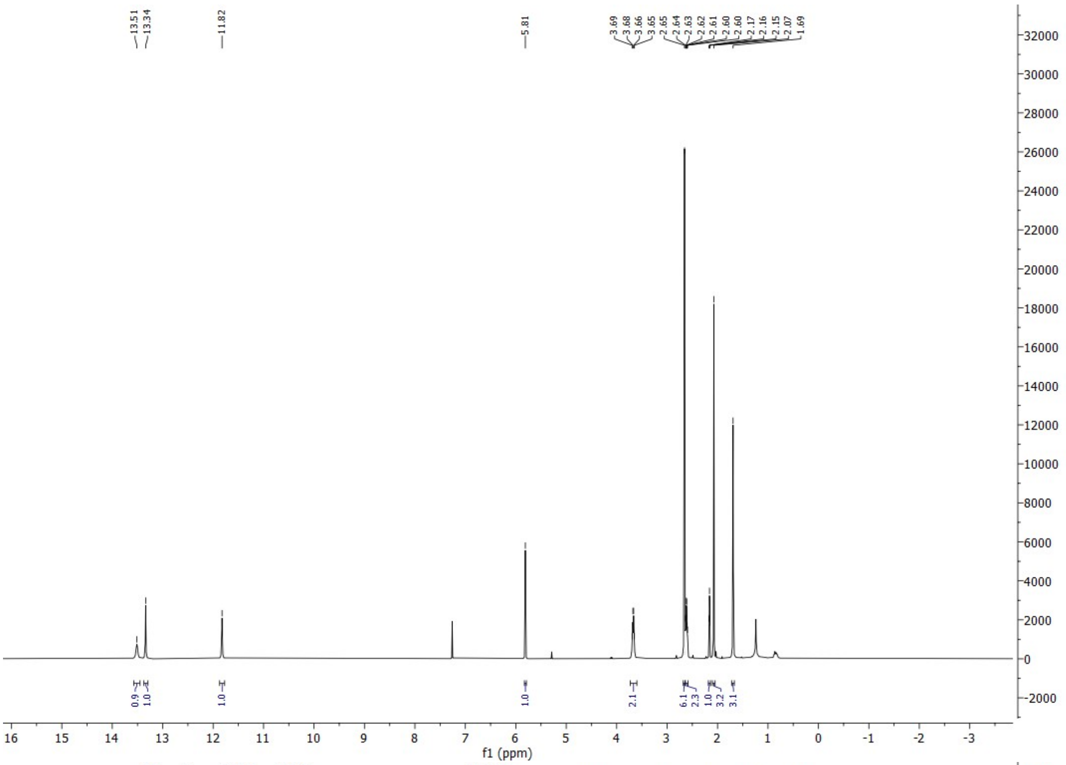

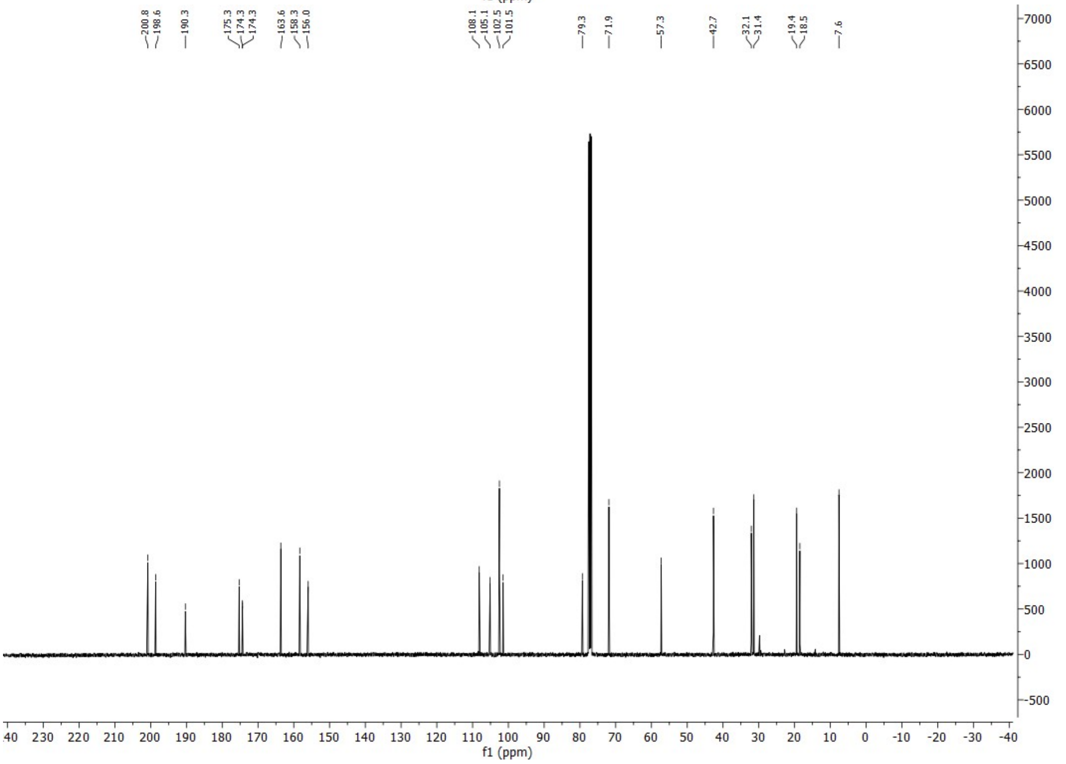

NMR-spectra of (*R*,*E*)-6-acetyl-2-(1-(but-3-yn-1-ylamino)ethylidene)-7,9-dihydroxy-8,9b-dimethyldibenzo[*b*,*d*]furan-1,3(2*H*,9b*H*)-dione (**3**). Top spectra is ^{1}H NMR (400 MHz, CDCl$_3$) and bottom is ^{13}C NMR (100 MHz, CDCl$_3$).

4

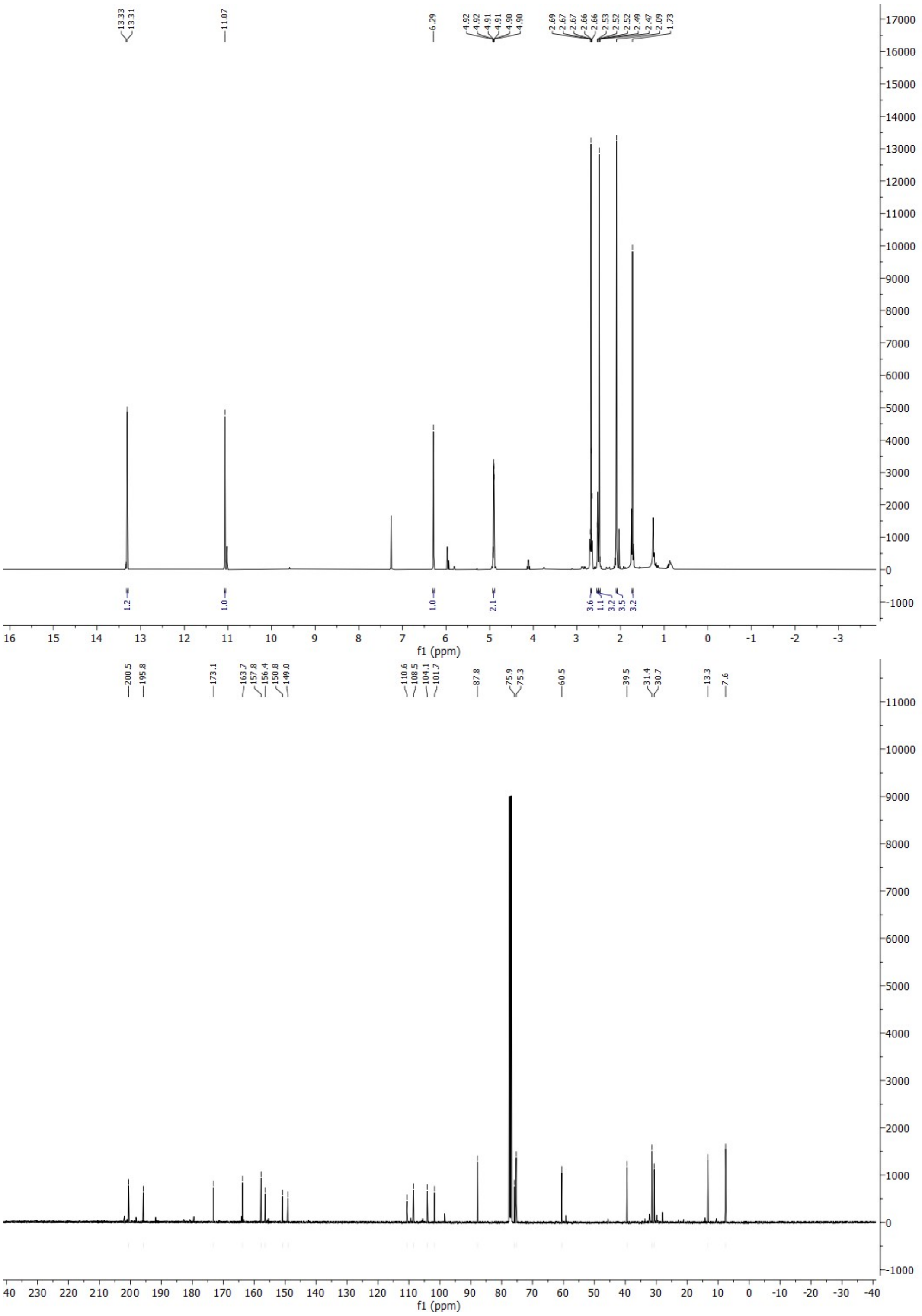

NMR-spectra of (*R*)-8-acetyl-5,7-dihydroxy-3,4a,6-trimethyl-1-(prop-2-yn-1-yl)-1,4a-dihydro-4*H*-benzofuro[3,2-*f*]indazol-4-one (**4**). Top spectra is ^{1}H NMR (400 MHz, $CDCl_3$) and bottom is ^{13}C NMR (100 MHz, $CDCl_3$).

5

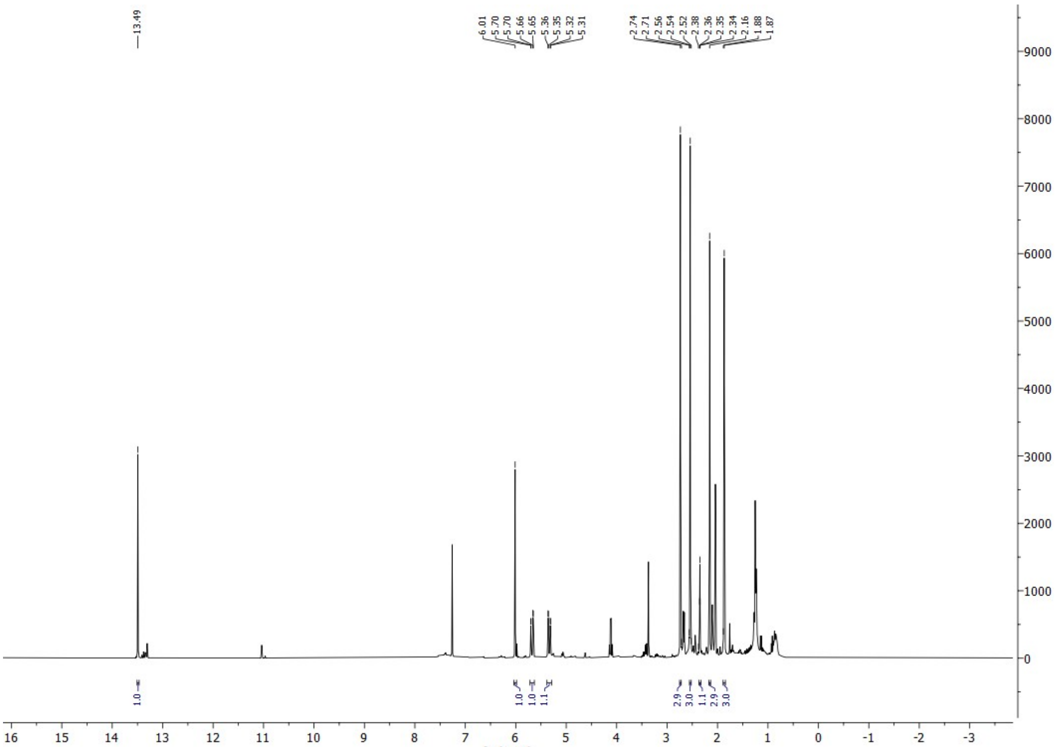

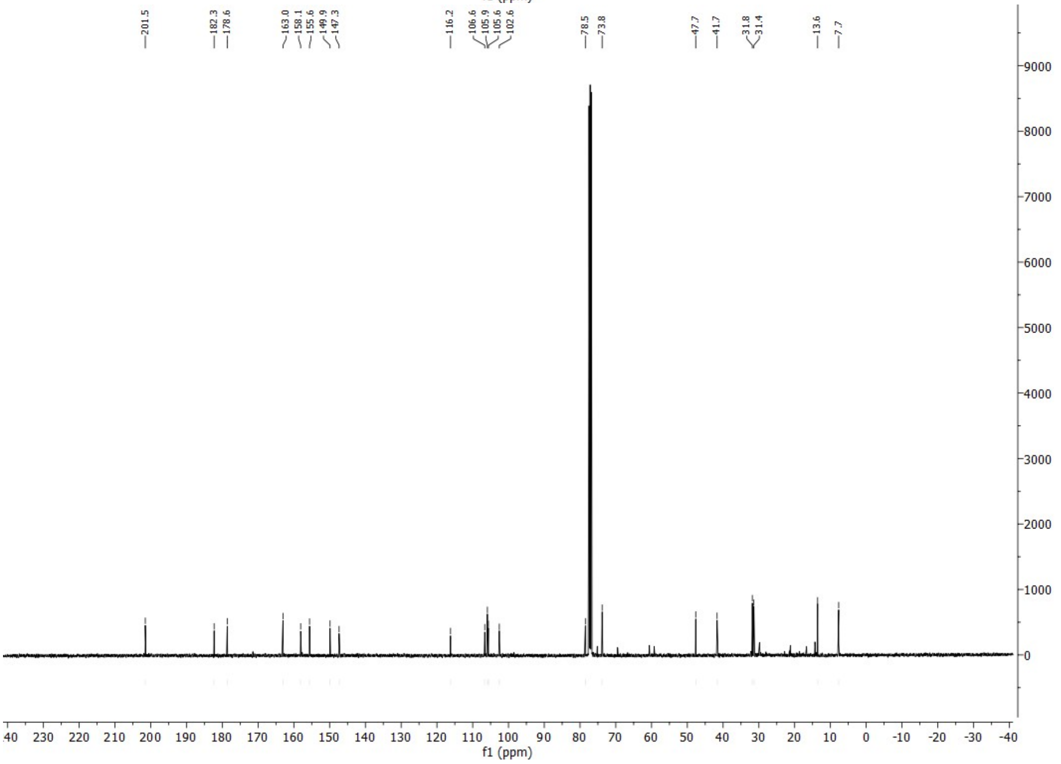

NMR-spectra of (*S*)-7-acetyl-8,10-dihydroxy-3,9,10b-trimethyl-1-(prop-2-yn-1-yl)-1,10b-dihydro-4*H*-benzofuro[2,3-*g*]indazol-4-one (**5**). Top spectra is ^{1}H NMR (400 MHz, $CDCl_3$) and bottom is ^{13}C NMR (100 MHz, $CDCl_3$).

References

[1] Y. S. Lo, W. H. Tseng, C. Y. Chuang, M. H. Hou, *Nucleic Acids Res.* **2013**, *41*, 4284–4294.

[2] K. Victor, L. Boris, G. Athina, P. Anthi, S. Marija, K. Marina, R. Oliver, S. Marina, *Medchemcomm* **2018**, *9*, 870–882.

[3] A. Pyrczak-Felczykowska, R. Narlawar, A. Pawlik, B. Guzow-Krzemińska, D. Artymiuk, A. Hać, K. Ryś, L. M. Rendina, T. A. Reekie, A. Herman-Antosiewicz, M. Kassiou, *J. Nat. Prod.* **2019**, *82*, 1768–1778.

[4] J. Cox, M. Mann, *Nat. Biotechnol.* **2008**, *26*, 1367–1372.

[5] J. Cox, M. Y. Hein, C. A. Luber, I. Paron, N. Nagaraj, M. Mann, *Mol. Cell. Proteomics* **2014**, *13*, 2513–2526.

[6] S. Tyanova, T. Temu, P. Sinitcyn, A. Carlson, M. Y. Hein, T. Geiger, M. Mann, J. Cox, *Nat. Methods* **2016**, *13*, 731–740.

[7] Y. Perez-Riverol, J. Bai, C. Bandla, D. García-Seisdedos, S. Hewapathirana, S. Kamatchinathan, D. J. Kundu, A. Prakash, A. Frericks-Zipper, M. Eisenacher, M. Walzer, S. Wang, A. Brazma, J. A. Vizcaíno, *Nucleic Acids Res.* **2021**, *50*, DOI 10.1093/nar/gkab1038.
